# Investigation of the direct effects of salmon calcitonin on human osteoarthritic chondrocytes

**DOI:** 10.1186/1471-2474-11-62

**Published:** 2010-04-05

**Authors:** Bodil-Cecilie Sondergaard, Suzi H Madsen, Toni Segovia-Silvestre, Sarah J Paulsen, Thorbjorn Christiansen, Christian Pedersen, Anne-Christine Bay-Jensen, Morten A Karsdal

**Affiliations:** 1 Nordic Bioscience A/S, Herlev Hovedgade 207, 2730 Herlev, Denmark; 2 Gentofte University Hospital Orthopedic Surgery Unit, 2820 Gentofte, Denmark; 3 Himmerland Hospital, Orthopedic Surgery Clinic, 9640 Farsoe, Denmark

## Abstract

**Background:**

Calcitonin has been demonstrated to have chondroprotective effects under pre-clinical settings. It is debated whether this effect is mediated through subchondral-bone, directly on cartilage or both in combination. We investigated possible direct effects of salmon calcitonin on proteoglycans and collagen-type-II synthesis in osteoarthritic (OA) cartilage.

**Methods:**

Human OA cartilage explants were cultured with salmon calcitonin [100 pM-100 nM]. Direct effects of calcitonin on articular cartilage were evaluated by 1) measurement of proteoglycan synthesis by incorporation of radioactive labeled ^35^SO_4 _[5 μCi] 2) quantification of collagen-type-II formation by pro-peptides of collagen type II (PIINP) ELISA, 3) QPCR expression of the calcitonin receptor in OA chondrocytes using four individual primer pairs, 4) activation of the cAMP signaling pathway by EIA and, 5) investigations of metabolic activity by AlamarBlue.

**Results:**

QPCR analysis and subsequent sequencing confirmed expression of the calcitonin receptor in human chondrocytes. All doses of salmon calcitonin significantly elevated cAMP levels (P < 0.01 and P < 0.001). Calcitonin significantly and concentration-dependently [100 pM-100 nM] induced proteoglycan synthesis measured by radioactive ^35^SO_4 _incorporation, with a 96% maximal induction at 10 nM (P < 0.001) corresponding to an 80% induction of 100 ng/ml IGF, (P < 0.05). In alignment with calcitonin treatments [100 pM-100 nM] resulted in 35% (P < 0.01) increased PIINP levels.

**Conclusion:**

Calcitonin treatment increased proteoglycan and collagen synthesis in human OA cartilage. In addition to its well-established effect on subchondral bone, calcitonin may prove beneficial to the management of joint diseases through direct effects on chondrocytes.

## Background

Osteoarthritis (OA) is the most common disease of the joints [1], and each year half-million Americans undergo total joint replacement [2]. Key characteristic of the disease are the accelerated degeneration of articular cartilage, changes in the matrix structure, and sclerosis of the subchondral bone. At present, there are no structure modifying drugs (SMOAD) accepted by either FDA (The US Food and Drug Administration) or EMEA (The European Medicines Agency). OA is a complicated condition of the entire joint, involving cartilage, bone, and the synovium, which highlights the complexity of the disease and may provide some understanding of why current treatments have not been successful [2-4].

The key components of articular cartilage are type II collagen and the proteoglycan aggrecan, which together constitutes 90% dry weight of healthy cartilage [5]. Current drug development strategies have particularly focused on inhibition of the enzymes responsible for degradation of these extra-cellular matrix (ECM) molecules, mainly the matrix metalloproteinases (MMPs) and the aggrecanases a disintegrin and metalloproteinase with thrombospondin motifs (ADAM-TS), which have resulted in potent MMP and aggrecanase inhibitors [6]. However, besides preclinical efficacy, these treatment opportunities have yet to demonstrate clinical efficacy and have additionally been implicated in the development of adverse musculoskeletal events [2].

Calcitonin is a 32-amino acid peptide hormone produced by the parafollicular cells in the thyroid gland that possess potent anti-resorptive effects by binding to its receptor on osteoclasts [7]. Calcitonin has been used in the treatment of osteoporosis (OP) for more than 30 years [8]. Various sources of calcitonin are found; of which salmon calcitonin is the most potent [7]. The effect of calcitonin on chondrocytes and cartilage metabolism is less investigated. Recently, it was proposed that articular chondrocytes express the calcitonin receptor and respond directly to calcitonin [9]. This has however been debated [10].

Salmon calcitonin has been shown to have chondroprotective effects in a range of animal models of OA. More specifically, calcitonin has been shown to counter the progression of joint lesions in a range of preclinical models, both traumatic and non-traumatic [11,12], suggesting potential chondroprotective effects. However, whether this apparent chondroprotective effect is mediated through modulation of subchondral bone turnover [10], either in part by effecting the peri-articular bone as suggested by Lin *et al*. and Behets *et al *[10,11] or as a direct effect of calcitonin articular cartilage, remains to be elucidated. A small number of investigators have focused their attention on the direct effects of calcitonin on articular cartilage of various species other than human, and even fewer has focused on cultured human OA chondrocytes *in vitro*, as summarized in [13]. The direct effect on human osteoarthritic articular cartilage with chondrocytes anchored in their natural environment remains to be addressed.

Articular cartilage explants have been shown to be a useful *in vitro *model of cartilage degradation and formation [14-18] where direct effects of new treatments on articular cartilage can be assessed. Compared to cultures of isolated chondrocytes, this model offers the advantage that the chondrocyte phenotype is preserved along with the intact ECM holding its natural integrins, inhibitors and growth factors, thus allowing for direct investigations of an *in vivo*-like situation. Applying the human articular cartilage explants model, we investigated the potential anabolic actions of calcitonin on the two major protein components in adult human OA articular cartilage, namely collagen type II and proteoglycans.

## Methods

All experiments were carried out using GLP and reagents of analytic grade. The human OA cartilage used for the experiments was obtained from female patients between the ages of 60-77 years undergoing total knee arthroplasty. The patients were informed by both oral and written communication at the hospital and gave their written consent before entering study, which was approved by the Capital Region of Denmark, DK-3400, approval number HD-2007-0084.

### Preparation of articular cartilage explants

Human OA articular cartilage from weight bearing uneroded areas with a normal smooth surface and hard texture was isolated and used for the experiments. Articular cartilage was cut into homologues explants, weighed (20 ± 2 mg wet weight) and placed in 96-well plates, and cultured in six-replicates under the following conditions at 37°C, 5% CO_2 _in serum-free medium D-MEM:F12 containing penicillin and streptomycin (Life Technologies, US), and to fortify the cultures in a controlled manner the medium was supplemented with 2% Ultrose G (Pall Life Sciences, FR). The cultures were repeated with five different patients on individual plates. The explants were cultured as follows: 1) in medium as control, 2) in medium with different doses of salmon calcitonin [0.1-100 nM] (Sigma-Aldrich, UK), 3) in medium with 100 ng/mL IGF (Sigma-Aldrich, UK) as positive control [19]. To represent the situation of complete metabolic inactivation, six explants were placed in cryo-tubes (Nunc, DK), frozen in liquid N_2_, and thawed at 37°C in water-bath for three repeated freeze-thaw cycles, allowing investigations of chondrocyte versus non-chondrocyte mediated effects. These explants were cultured in medium only. The conditioned medium was replaced every 2^nd ^to 3^rd ^day for sixteen days and the collected conditioned medium was stored frozen at -20°C until further analysis.

### Investigation of cell viability

Cell viability was examined on the last day of culture using the AlamarBlue colorimetric assay (Invitrogen, DK). In brief, AlamarBlue solution was diluted to 10% with the respective refreshed treatments of the cartilage explants and incubated with the cartilage explants for three hours. The fluorescence was measured at 540 nm and 650 nm.

### Radioactive labeling by ^35^SO_4_

Formation of proteoglycans was followed by radioactive-labeled precursor incorporation of ^35^S as liquid sulfate (Amersham Biosciences, US). On day sixteen of culture, explants were pulsed with 5 μCi ^35^SO_4 _added to the refreshed conditioning medium to each culture and incubated for 24 hours more. After pulsing, the explants were washed 5 times with PBS and the explants were dissolved for 48 h at 2-8°C in 4 M GuHCl (buffer: 50 mM sodium acetate, pH 5.8, containing 10 mM EDTA and 0.1 M caproic acid) to release all soluble proteoglycans like chondrotin sulphate and keratin sulphate side chains [20]. The incorporation of radioactive-labeled ^35^SO_4 _was quantified independently for each proteoglycan extract of the cartilage explants samples using liquid scintillation counting and normalized to the amount of cultured cartilage.

### PIINP, biochemical marker of collagen type II formation

The quantification of pro-peptides from the N-terminal of collagen type II (PIINP) by ELISA (IDS Ltd., UK) was used for the assessment of collagen type II formation from the conditioned medium at day 16 of culture. The PIINP ELISA is a competitive enzyme-linked immunoassay based on a monoclonal antibody (F7504) recognizing a linear sequence GPQGPAGEQPGRGDR located in the center of the N-terminal pro-peptide of collagen type II [21].

### RNA extraction

The human articular cartilage (150 mg wet weight) was obtained in RNALater (Applied Biosystems, UK) directly from the operation theater and pulverized in liquid nitrogen by the Bessman Tissue Pulverizer (Spectrum Laboratories, US). Lysis of cells and RNA extraction was performed using the PicoPure™ RNA Isolation Kit (Molecular Devices, US). On column DNase digestion was performed for 7.5 minutes with DNA-free (Ambion, UK). Human osteoclasts were retrieved as described previously [9,22], and the RNA purified using the RNA isolation kit (Qiagen, UK). The RNA was quantified using a NanoDrop-1000 Spectrophotometer (Thermo Fisher Scientific Inc, US).

### cDNA synthesis and QPCR

cDNA for QPCR was prepared using Transcriptor First Strand cDNA Synthesis Kit (Roche Diagnostics, US) with anchored-oligo (dT)_18 _primer and random hexamer primers. No template controls (NTC) were prepared without the addition of reverse transcriptase to the cDNA synthesis. QPCR was performed on a Stratagene Mx3000Pro using Brilliant SYBR Green II Mastermix (Stratagene Agilent Technologies, US). SYBR Green melting temperatures (T_m_) of the products were determined for all calcitonin receptor primer pairs by QPCR analysis on a plasmid encoding the entire calcitonin receptor. The calcitonin receptor primers were designed to the coding region of the calcitonin receptor from the accession number NCBI: [NM_001742] and primers of SURF1 was used as a housekeeping gene, see table 1 for primer details. Primer pairs were used in concentrations of 200 nM, and all analysis were performed in duplicates. Validity of QPCR products were confirmed based on SYBR green melting curves, visualization on a 2% agarose gel stained with ethidium bromide, and finally sequencing of calcitonin receptor products (Eurofins MWG Operon, DE).

**Table 1:** Location of primerpairs in the coding region of the human calcitonin receptor.

Gene	Forward	Reverse	Product length	**Product T**_**m**_
**SURF1**	5-CAGCAGACCCAATGGAAC-3	5-CCAGGATGGTGACTCCC-3	238 bp	~83.6
**Coll2A**	5-AGGATGGTGACTCCCAAG-3	5-GTCCATGGGTGCAATGTCAA-3	136 bp	~83.5
**CalcR 1**	5-GACAACTGCTGGCTGAGTG-3	5-GAAGCAGTAGATGGTCGCAA-3	321 bp	~84.1
**CalcR 2**	5-TGATTCATTTCCAGGGCTTC-3	5-GCTGGTTCATTCCTCAGCTC-3	220 bp	~88.2
**CalcR 3**	5-GGTAACCCTGCACAAGAAC-3	5-AAGCGTTGCTTCTCAGTAAA-3	235 bp	~82.0
**CalcR 4**	5-ACACCGGCTGGAGTATTGTC-3	5-AAAATCCCCAGGGAAATCAC-3	257 bp	~79.8

Housekeeping gene: SURF1; Collagen type II: Coll2A; Calcitonin receptor 1-4: CalcR 1-4.

Length of PCR products, base pairs (bp) and melting temperature (T_m_)

### Intracellular signaling

#### Response to calcitonin

Human OA articular cartilage was obtained from patients undergoing total knee arthroplasty from the patient group mentioned above. Articular chondrocytes were isolated from the ECM by enzymatic digestion with 0.5% trypsin (Sigma-Aldrich, UK) for 30 minutes and 0.5% collagenase (Wako, DE) for 4 hours at 37°C. Chondrocytes were obtained after filtration and centrifugation at 1600 × *g *for 10 min. The pellet was re-suspended in α-MEM containing 10% foetal bovine serum (FBS) (Sigma-Aldrich, UK), and sub-cultured for four days at 37°C, 5% CO_2_. The cells were lifted, centrifuged, and cultured under serum-free conditions for 24 hours, and subsequently pre-inhibited for one hour with 100 mM 3-isobutyl-1-methylxanthine, IBMX (Sigma-Aldrich, UK), and afterwards stimulated with 1 pM-100 nM salmon calcitonin for 15 minutes. IBMX [100 mM] was used alone as a background control. Quantification of the intracellular second messenger cAMP was performed according to the EIA kit protocol (Amersham Biosciences, US).

After the first passage and centrifugation the chondrocytes regain their spherical appearance. In order to confirm that the isolated chondrocytes still expressed the calcitonin receptor, immunocytochemistry was performed using mouse calcitonin receptor antibody directed towards the human calcitonin receptor (AbD Serotec, UK) on chondrocytes cultured in 96-well plates parallel to the studies. Chondrocytes with a fibroblast-like appearance did not react with the calcitonin receptor antibody, whereas all the spherical cells did, these results are not shown.

#### Antagonist competition binding

Chondrocytes were retrieved as described above. The chondrocytes were seeded at a density of 40.000 cells/well in 96-well plates, under serum-free conditions and incubated at 37°C, 5% CO_2_. The next day, all primary chondrocytes were stimulated with 100 mM IBMX for one hour. Subsequently, some cells were pre-incubated with 100 nM the calcitonin receptor antagonist 8-32 sCT (Bachem, CH) + 100 mM IBMX for 30 minutes. Other cells had refreshment of 100 mM IBMX for reference. Subsequently the antagonist treated cells were incubated with 100 nM salmon calcitonin for 30 min. As a positive control, 100 μM forskolin, an activator of adenylylcyclase, was used. In all instances the supplemented medium was preheated and the treatments balanced in concentration so that only 50% of the medium was changed during addition of the IBMX, antagonist or salmon calcitonin. After the last incubation, the cells were subjected to lysis and the cAMP levels were quantified as described above.

### Statistics

Results are shown as mean + SEM. All *in vitro *experiments were conducted with a minimum of five repetitions and all explants cultures in 6 replicates for each treatment. The results shown from biochemical markers and incorporation are representative from one subject, a 78 year old female, and comparable results were obtained from all subjects. Differences between mean values were compared group by group using Student's two-tailed t-test for unpaired observations using the GraphPad Prism software and under assumption of normal distribution. Differences were considered statistical significant if P < 0.05.

## Results

### Calcitonin stimulates proteoglycan synthesis in human articular cartilage explants

**Figure 1:**
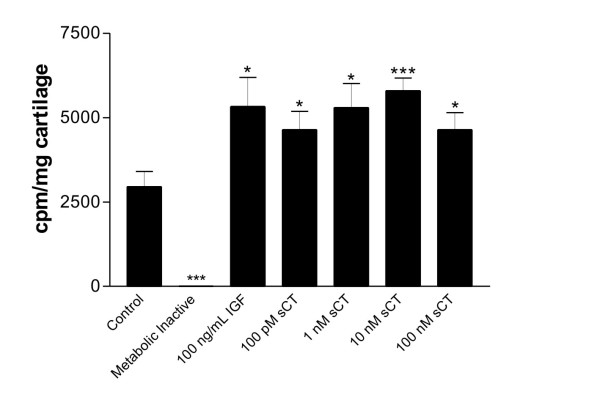
**Calcitonin stimulates proteoglycan formation in human osteoarthritic cartilage**. Human OA articular cartilage explants were cultured with or without doses of salmon calcitonin, and on day 16 pulsed with 5 μCi ^35^SO_4 _for 24 hours. The proteoglycans were extracted and the incorporation of ^35^SO_4 _were followed by liquid scitillation counting. Insulin-like growth factor (IGF) was used as a positive control to induce formation in articular cartilage. The bars represent the mean value of count pr. minute, cpm, from 6-replicates and the indivdual values were adjusted for the amount of cultured cartilage. The errorbars are the standard error of mean, SEM. The asterisks symbolize the levels of statistical difference to the vehicle control, * P < 0.05, ***P < 0.001.

The effects of salmon calcitonin on human articular cartilage proteoglycan synthesis were investigated in response to different doses (100 pM-100 nM). Figure 1, shows the proteoglycan synthesis under the different conditions as determined by the levels of incorporated ^35^SO_4_. Significantly more sulfate was incorporated into the papain extractable proteoglycans of the control explants compared to metabolic inactive, non-cell-mediated background levels P < 0.001. The 10 nM salmon calcitonin significantly induced a 96%, (P < 0.001), increase in proteoglycan synthesis. All other concentrations, including the very low 100 pM concentration, P < 0.05, increased levels incorporated ^35^SO_4 _compared the vehicle control. IGF was included as a positive control, which induced an 80% (P < 0.05) increase in proteoglycan synthesis.

### Calcitonin stimulates cartilage formation measured by the release of pro-peptides of collagen type II

**Figure 2:**
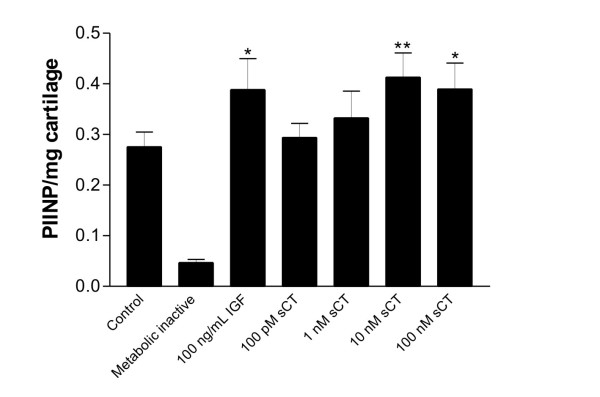
**Stimulation of human OA cartiage explants with salmon caltionin increase the release of pro-peptides of type II collagen**. Human OA cartilage explants were cultured for 16 days with different doses of salmon calcitonin and IGF as positiv control. The released neoepitopes in the supernatant from day 16, were quatifiened by an ELISA detecting the N-terminal collagen type II pro-peptides, PIINP. The bars represent the mean value from 6-replicates and the indivdual values were adjusted for the amount of cultured cartilage. The errorbars are the standard error of mean, SEM. The asterisk symbolize the levels of statistical difference to the vehicle control, * P < 0.05. **P < 0.01.

To further assess the effect of salmon calcitonin on collagen type II synthesis, the levels of PIINP in the conditioned medium was quantified. As expected, the positive control significantly increased the levels of PIINP (P < 0.05), see figure 2. Doses of 10 and 100 nM salmon calcitonin significantly stimulated the release of pro-peptides of collagen type II measured by the PIINP ELISA compared to explants cultured in medium only, P < 0.01 and P < 0.05, respectively.

### Human OA articular chondrocytes express the calcitonin receptor

**Figure 3:**
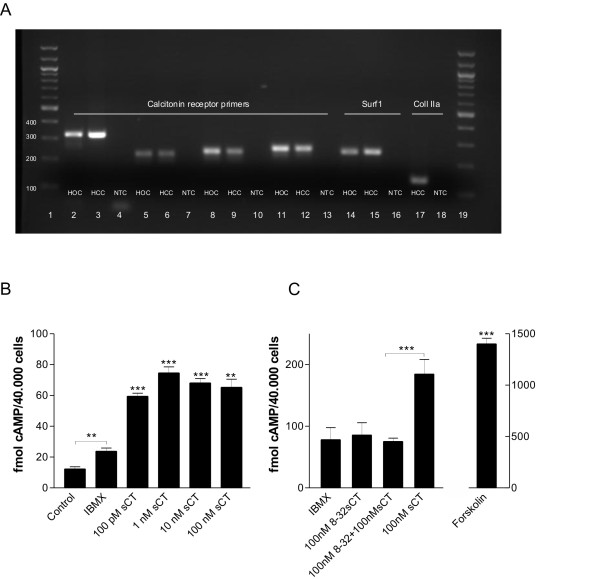
**Human chondrocytes express the calcitonin receptor**. **A**. Calcitonin receptor expression in human osteoclasts and cartilage was analyzed using QPCR. QPCR products were visualized on a 2% agarose gel. Lanes: 1+19 NEB 100 bp DNA ladder, 2-4 CalcR1, 5-7 CalcR2, 8-10 CalcR 3, 11-13 CalcR 4, 14-16 Surf 1, 17-18 Coll2a. Abbreviations: CalcR - calcitonin receptor primer pair 1-4; Coll2a - Collagen type II; HOC - human osteoclasts; HCC - human chondrocytes; NTC - no template control. **B**. Intracellular cAMP levels after stimulation with salmon calcitonin: Human OA chondrocytes were stimulated with IBMX for one hour, followed by 15 minutes stimulation in presence or absence of salmon calcitonin as specified in the figure legends. **C**. Binding competition by the calcitonin receptor antagonist 8-32sCT. Forskolin was used as positive control. The *asterisks *indicate significant differences (** P < 0.01, *** P < 0.001).

The expression of calcitonin receptor mRNA from human articular chondrocytes (HCC) was investigated by QPCR using four different calcitonin receptor primer pairs designed to be intron spanning and to cover different areas of the coding region of the human calcitonin receptor. As a positive control, the expression of calcitonin receptor was analyzed in human osteoclasts (HOC), and as negative controls NTC's were included. The expression of the housekeeping gene Surf1 was analyzed in both HCC and HOC, and the expression of the HCC-specific gene collagen type IIa was investigated in the HCC. QPCR analysis using the four different calcitonin receptor primer-pairs showed the presence of the calcitonin receptor mRNA in HCC in addition to the HOC. The QPCR results were confirmed by sequencing of the PCR products. Figure 3A shows a representative image of the QPCR amplicons.

### Calcitonin signals through the cAMP pathway

Signaling of calcitonin through the calcitonin receptor and binding to G-coupled receptors has previously been shown to activate adenylylcyclase, resulting in increased cAMP levels [23]. The response of cAMP levels to salmon calcitonin stimulation of isolated HCC was investigated. The chondrocytes were exposed to salmon calcitonin in co-culture with [100 μM] IBMX, a non-specific inhibitor of cAMP phosphodiesterases, hereby preventing the cyclic nucleotide hydrolysis. Salmon calcitonin significantly induced levels of cAMP in articular chondrocytes, see figure 3B. The concentrations of salmon calcitonin caused a more than 100% increase in cAMP activation. A maximal >200% induction in cAMP activation was observed by the [1 nM] dose (P < 0.001) compared to [100 μM] IBMX.

Furthermore, blocking of the cAMP signaling was investigated by competition of receptor binding by the calcitonin receptor antagonist 8-32sCT. Binding of the 8-32sCT do not lead to a response in the second messenger cAMP. Addition of the antagonist together with the highest dose of salmon calcitonin (100 nM) caused a 50% (P < 0.001) reduction compared to calcitonin alone, see figure 3C. The cAMP release was comparable to IBMX levels when the antagonist was added to cultures with or without salmon calcitonin (100 nM). The positive control forskolin significantly increased cAMP levels (P < 0.001).

### Cell viability in response to salmon calcitonin

**Figure 4:**
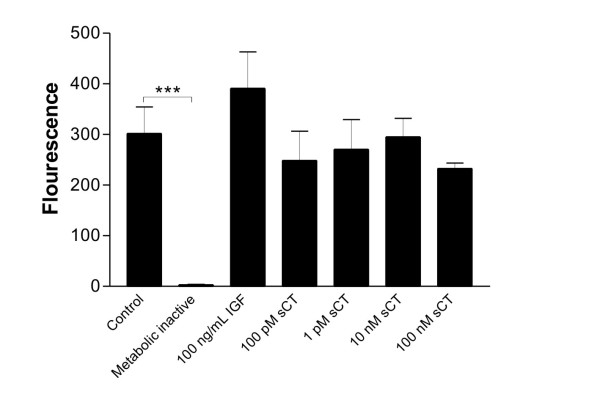
**Chondrocyte viability**. The chondrocyte viability was investigated by the metabolic dye AlamarBlue and was by quantified by fluorescence. The bars represent the mean value from 6-replicates at the last day of culture and the indivdual values were adjusted for the amount of cultured cartilage. The errorbars are the standard error of mean, SEM, and the *asteriks *indicate ***, P < 0.001. Salmon calcitonin: sCT.

To investigate whether salmon calcitonin has an effect on cell number and metabolic activity, cell viability were investigated by AlamarBlue in the explants culture at the last day of culture. As the cells metabolize, the redox-potential changes, which makes the metabolic dye change color from blue to purple, thus allowing the viability of the cells to be by quantified by fluorescence. The fluorescence levels from metabolic inactivated cartilage corresponds to the background levels and was significantly different from the control, P < 0.001, as seen in figure 4. The treatments with salmon calcitonin did not affect the cell viability significantly and thus the salmon calcitonin not toxic to the chondrocytes. IGF did not affect chondrocyte cell viability.

## Discussion

These current data highlight that salmon calcitonin concentration-dependently and significantly stimulated proteoglycan and collagen type II synthesis in human osteoarthritic cartilage *in vitro *and validates that human OA chondrocytes express the calcitonin receptor. Salmon calcitonin has been demonstrated by different research groups to have chondroprotective effects in a range of animal models of OA, representing different aspects of the disease. However, in respect to the documented preclinical efficacy, the precise mode of action still remains to be evaluated.

The current experiments have provided evidence for a potential anabolic effect of calcitonin on human osteoarthritic chondrocytes, both with respect to proteoglycan and collagen type II synthesis. We used IGF as a positive control that previously has been shown to stimulate cartilage formation [19]. The biochemical marker PIINP was used for investigation of the direct effect of salmon calcitonin on collagen type II formation [21]. Salmon calcitonin stimulated cartilage synthesis to the same level as the positive control, IGF. However, these data highlight the small window of response in the current line of experiments, and the smaller responsiveness of old human articular cartilage [24] compared to that of young bovine cartilage or other types of cartilage as previously observed [25]. We investigated proteoglycan synthesis by the gold standard method, radio-labeled incorporation of ^35^SO_4_. We found that salmon calcitonin dose-dependently increased proteoglycan incorporation into the matrix. These data are in alignment with those presented by other groups, although the results are from non-human models [12,26-30].

In the present studies we focused on the direct effect of salmon calcitonin on human osteoarthritic cartilage, as the effect on bone resorption and remodeling are well understood and accepted [31]. We employed an articular cartilage explants model in which the chondrocytes are embedded in their natural matrix environment, which preserves chondrocyte phenotype [32]. This may provide a different phenotype of chondrocytes compared to that of growth plate and mono-layers of proliferating chondrocyte-like cells. It is becoming more and more evident that the ECM genotype modulates the phenotype of cells [33] and by that the cellular response to different stimuli.

Calcitonin has on previous occasions been shown to have direct anabolic effects on cartilage, however this was limited to hypertrophic chondrocytes in growth plate cartilage of rats [29,34], and other development cartilage forms in avian models [28,30,35]. Kato *et al*. [36] demonstrated that calcitonin stimulated the sulfation of proteoglycans in isolated chondrocytes from rats and rabbits. The response to calcitonin was investigated in human chondrocytes by Franchimont [26], albeit this was done in isolated chondrocytes cultured in clusters without their ECM. Human articular chondrocytes from OA patients may be different compared to that of development models that includes, but are not limited to composition of the matrix, age of the investigated cells and natural differences among species. The current data further aid in the understanding of the anabolic effects of salmon calcitonin.

Many findings in the literature point toward a pharmacological effect of calcitonin on chondrocytes [26,35,37], but the response may not exclusively be mediated by the calcitonin receptors. The calcitonin receptors are promiscuous with respect to ligand binding. Thus, calcitonin, calcitonin gene-related peptide (CGRP), adrenomedullin (AM) and amylin (AMY) are able to displace each other from specific binding sites, implying significant cross-reactivity of each of them with the receptors of other peptides. Receptor activity-modifying proteins (RAMPs) are single transmembrane proteins that are critical to the calcitonin family G-protein coupled receptors for receptor-ligand recognition. The calcitonin receptor has 60% homology to the calcitonin receptor-like receptor (CL), and the calcitonin receptor predominantly recognizes calcitonin in the absence of RAMPs. Alpha-CGRP, AM, and AMY are also able to signal through the calcitonin receptor [38]. In the presence of RAMP 3, the calcitonin receptor only interacts with AMY [39]. An AMY/CGRP receptor is recognized when a calcitonin receptor is co-expressed with RAMP 1. RAMP 1-transported CL is a CGRP receptor. RAMP 2- or RAMP 3-transported CL are AM receptors [40]. The CL is identified as a CGRP receptor when co-expressed with RAMP 1. The same receptor is specific for AM in the presence of RAMP 2. Thus, due to the complex promiscuous properties of the calcitonin family receptors it is not possible from the current experiments to rule out signaling of salmon calcitonin through the other receptors.

We indirectly investigated the binding of salmon calcitonin to the calcitonin receptor by examining the levels of intracellular cAMP in isolated chondrocytes and thereby the functionality of the receptor. Additionally we show data that indicates that the cAMP induction of salmon calcitonin could be completely blocked by an antagonist to the calcitonin receptor, 8-32sCT. G-coupled 7TM receptors have cAMP as a second messenger and calcitonin is known to increase cAMP levels in other cell types [23]. Our group has recently showed that cAMP is an important determinant in directing chondrocytes from a catabolic to an anti-catabolic shift in phenotype [25]. These data are in alignment with other findings suggesting cAMP may be part in an important shift in chondrocyte phenotype [41,42]. These findings are in alignment indicating that elevated levels of cAMP play an important part in the shift into a more cartilage producing anabolic cell phenotype. However, this smaller elevation of cAMP may only be an intermediate factor in the complex machinery of cellular signaling pattern eventually leading to cartilage synthesis. The cellular actions remain to be investigated and are beyond the scope of this report.

The present data may be in alignment with those of previous investigators, but not with the findings by Lin *et al*. [10]. A recent report from the group showed that the calcitonin receptor was not expressed in human chondrocytes. However, this central contradiction may be explained by important experimental differences. In the current experiments mRNA was obtained from freshly isolated articular cartilage, collected in RNALater at the operation theater. In contrast, the chondrocytes used by Lin *et al*. were not primary chondrocytes but chondrocytes cultured in 20% serum until confluence, a treatment known to change the chondrocyte phenotype and make the cells dedifferentiate to a fibroblast-like cell type and hence effect the gene expression. In disagreement with the report by Lin *et al*. we show the presence of this particular fragment in articular chondrocytes and additionally by three other primer pairs. The difference in these findings may be due to QPCR sensitivity issues taking into account the low expression of the calcitonin receptor in adult human cartilage. As pointed out by the group, the expression of the calcitonin receptor may indeed be regulated by age, but may also be influenced by the location in the cartilage matrix. Other research groups have found the receptor in hypertrophic deep zones of the cartilage in pre-clinical models [29]. We exclusively used articular cartilage from the surface layer of the femur plateau. In addition, there might be differences in the expression of the calcitonin receptor in healthy cartilage compared to cartilage from OA patients. Further research may be needed to understand the differences of expression patterns of chondrocytes that may be highly related to their immediate phenotype. These subpopulation of chondrocytes includes but are not limited to those affected by age, sex, clinical history, and whether chondrocytes are hypertrophic, proliferating, matrix synthesizing and in addition originate from weight bearing or non weight bearing areas. The presented data showed expression of the calcitonin receptor at RNA-levels. However in a previous publication we have also shown the expression of the calcitonin receptor at protein levels by immunohistochemistry in the upper zones of human OA articular cartilage with the chondrocytes embedded in the matrix [9].

Our investigations did not set out to quantitatively evaluate the amount of the calcitonin receptor, but merely to investigate its expression in human articular chondrocytes. However, comparison between the expression of the housekeeping gene Surf1 and that of all calcitonin receptor amplicons based purely on Ct values (~30 vs. ~36) clearly showed that the calcitonin receptor is expressed in low levels. Thus, to ensure QPCR detection, the method must be optimized, which we obtained by use of a calcitonin receptor expressing plasmid. Accordingly, we determined all SYBR Green melting temperatures for the calcitonin receptor primer pairs by QPCR analysis on a plasmid encoding the entire calcitonin receptor. In the present study we investigated the calcitonin receptor expression in human articular cartilage isolated directly without any prior culturing. We have likewise examined the expression of the calcitonin receptor using the same four primer pairs and additionally in pronase- and collagenase-retrieved isolated human chondrocytes pooled from four patients, and were in all instances able to detect calcitonin expression (data not shown). Of concern, multiple splice variants of the calcitonin receptor exists in the same organism, and at least four are recognized in humans, of which one is located in the non-coding region of the receptor [43,44]. Further research is needed to investigate which splices variant or variants as well as their signaling in human articular chondrocytes.

As mentioned in the introduction, OA involves changes in the joint triadic: bone, cartilage and synovium. It is still debated, which parameters are the initiators or drivers of disease progression, although it is becoming more clear that inflammation may not initiate the disease but rather play an important role in the progression of the disease at later stages [4]. Intuitively the chondroprotective effects of calcitonin may in part be mediated through a direct action on chondrocytes and in part through effects on subchondral bone remodeling [4]. We have previously, under *in vitro *conditions, shown that salmon calcitonin was able to decrease cytokine-stimulated degradation in bovine articular cartilage explants in connection to lowering of the proteolytic activity and expression of MMPs [9]. The anti-catabolic actions of calcitonin have been observed in isolated human chondrocytes cultured in clusters by Franchimont *et al*. [26]. Some researchers have found anti-inflammatory effects of calcitonin, albeit these effects remain to be further understood in relation to clinical OA [45,46].

The putative effect of salmon calcitonin on the progression of OA has been investigated under clinical settings and focused on the positive effects on cartilage degradation [47,48]. In the context of the current data in, which positive effects of cartilage formation was observed, future studies might be undertaken to investigate the effect on cartilage synthesis markers such as PIINP or PIIAnP and thereby more optimally estimate possible efficacy [32,47].

## Conclusion

Calcitonin treatment increased proteoglycan and collagen synthesis of human OA cartilage. Calcitonin may provide additional positive effects on joint health directly on articular chondrocytes in addition to the well established osteoclast mediated effects on subchondral bone.

## Abbreviations

ADAM-TS: A disintegrin and metalloproteinases with thrombospondin motifs; AM: Adrenomedullin; AMY: Amylin; CGRP: Calcitonin gene-related peptide; CL: Calcitonin receptor-like receptor; ECM: Extra-cellular matrix; EMEA: The European Medicines Agency; FDA: The US Food and Drug Administration; HCC: Human chondrocytes; HOC: Human osteoclasts; IGF: Insulin-like growth factor; MMP: Matrix metalloproteinases; NTC: Negative controls/No template control; OA: Osteoarthritis; OP: Osteoporosis; PIINP: Pro-peptides from the N-terminal of collagen type II; PIIAnP: Pro-peptides from the N-terminal of collagen type IIA; RAMP: Receptor activity-modifying proteins; sCT: Salmon calcitonin; SEM: Standard error of mean; SMOAD: Structure modifying drugs; TNF: Tumor necrosis factor alpha.

## Competing interests

BC Sondergaard, SH Madsen, A Segovia, SJ Paulsen, C Christiansen, AC Bay-Jensen, and MA Karsdal are employees at Nordic Bioscience. MA Karsdal and C Christiansen hold stocks in Nordic Bioscience A/S. All other authors have no conflicts of interest.

## Authors' contributions

BCS did the writing of the manuscript and performed all cultures and biochemical analysis, and SHM assisted the cAMP analysis. AS and SJP designed the primers and performed most of the molecular biology. CC and MAK were involved in designing the experiments and interpretation of the results. TC informed the patients and collected the written consent and all the biological material. All authors reviewed the manuscript. All authors read and approved the final manuscript.

## Pre-publication history

The pre-publication history for this paper can be accessed here:

http://www.biomedcentral.com/1471-2474/11/62/prepub
